# Influence of prior anterograde shear rate exposure on exercise-induced brachial artery dilation

**DOI:** 10.14814/phy2.12414

**Published:** 2015-05-25

**Authors:** Carl J Ade, Michael G Brown, Austin K Ederer, Rachel N Hardy, Landon K Reiter, Kaylin D Didier

**Affiliations:** Department of Health and Exercise Science, The University of OklahomaNorman, Oklahoma

**Keywords:** Blood Flow, exercise, shear rate

## Abstract

Shear rate can elicit substantial adaptations to vascular endothelial function. Recent studies indicate that prior exposure to anterograde flow and shear increases endothelium-dependent flow-mediated dilation at rest and that anterograde shear can create an anti-atherosclerotic and provasodilatory state. The primary aim of the present study was therefore to determine the effects of prior exposure to anterograde shear on exercise-induced brachial artery dilation, total forearm blood flow (FBF), and vascular conductance (FVC) during dynamic handgrip exercise. Eight men completed a constant-load exercise test corresponding to 10% maximal voluntary contraction, prior to (baseline) and following a 40 min shear rate intervention (post-SRI) achieved via unilateral forearm heating, which has previously been shown to increase anterograde shear rate in the brachial artery. During the SRI, anterograde shear rate increased 60.9 ± 29.2 sec^−1^ above baseline (*P* < 0.05). Post-SRI, the exercise-induced brachial artery vasodilation was significantly increased compared to baseline (4.1 ± 0.7 vs. 4.3 ± 0.6 mm, *P* < 0.05). Post-SRI FBF mean response time (33.2 ± 16.0 vs. 23.0 ± 11.8 sec, *P* < 0.05) and FVC mean response time (31.1 ± 12.8 20.2 ± 10.7 sec, *P* < 0.05) at exercise onset were accelerated compared to baseline. These findings demonstrate that prior exposure to anterograde shear rate increases the vascular responses to exercise and supports the possible beneficial effects of anterograde shear rate in vivo.

## Introduction

Mechanical shear stress acting on the endothelium is a key stimulus mediating vascular adaptation (Tinken et al. 2009; Newcomer et al. 2011). Evidence from isolated cell culture studies suggests that endothelial cells exposed to an increased shear stress exhibit a cascade of increased intracellular signaling which could contribute to adaptations in vascular function (Laughlin et al. 2008). A sustained increase in shear stress results in endothelial adaptations. Following 3 h of exposure to unidirectional shear stress, cultured endothelial cells increase endothelial nitric oxide synthase (eNOS) gene transcription and eNOS mRNA expression (Ranjan et al. 1995; Uematsu et al. 1995). Similarly, 2 h of elevated shear stress in isolated perfused coronary arterioles increases eNOS and super oxide dismutase (SOD-1) expression resulting in an increased NO bioavailability (Woodman et al. 1999). Recently, Tinken et al. (2009) increased mean shear rate in the human forearm using a novel forearm heating protocol and demonstrated that following exposure to elevated anterograde shear rate brachial artery endothelial function is significantly increased (2009). Cumulatively, these data suggest that acute elevations in shear rate result in an enhanced endothelial function. While the original work of Tinken et al. (2009) suggests that prior exposure to anterograde shear rate increases resting endothelial function (2009), it remains unknown whether the vascular responses to dynamic exercise, which are also dependent on endothelial function (Wray et al. 2011), are also improved. Therefore, the primary aim of the present study was to determine if prior increased anterograde shear stress in a conduit artery increases exercise-induced brachial artery dilation in response to moderate intensity dynamic handgrip exercise. It was hypothesized that prior acute exposure to anterograde shear via forearm heating, similar to previous investigations (Tinken et al. 2009; Naylor et al. 2011), would (1) accelerate the blood flow and vascular conductance responses to dynamic forearm exercise and (2) increase the exercise-induced brachial vasodilation.

## Methods

### Subjects

Eight men (24.3 ± 3.9 years (mean ± SD)) completed the study. All subjects were free from known cardiovascular, pulmonary, or metabolic disease and were nonsmokers as determined via health history questionnaire. All subjects were untrained and participated in less than 150 min of moderate intensity or 75 min of vigorous physical activity on at least 3 days of the week. Verbal and written consent were obtained following approval of the study by the Institutional Review Board for Research Involving Human Subjects at the University of Oklahoma, which conformed to the Declaration of Helsinki. Testing sessions were performed in a temperature-controlled laboratory (21–22°C). Each subject reported to the laboratory in a fasted state and after refraining from exercise, alcohol, and caffeine for at least 12 h.

### Forearm handgrip exercise

Maximum isometric handgrip contraction strength (MVC) was measured in triplicate in the right arm with a handgrip dynamometer to determine a reference value. Following a 15 min rest period, forearm handgrip exercise on a custom-built ergometer was performed at 10% MVC for 6 min to ensure a steady state was achieved. Ten percent MVC was chosen to minimize any residual effects that might be evident with higher intensity work rates. The work rate was achieved by lifting a weight ~3 cm over a pulley at a duty cycle of 1 sec contraction and 2 sec relaxation, with a metronome used to ensure correct timing. The exercise test was performed before (baseline) and 15 min after the heating shear rate intervention (post-SRI).

### Shear rate intervention (SRI)

Acute adjustment of the shear rate pattern in the brachial artery of the right arm was performed between forearm handgrip exercise tests in the supine position via unilateral forearm heating in a similar method as Tinken et al. (2009). Briefly, the right hand and forearm were enclosed in a water-perfused sleeve, which covered the entire lower arm. The sleeve was perfused with warm water (41°C) for 40 min. The water temperature was maintained constant at 41 ± 1°C via a thermostatically controlled heating unit with a water storage reservoir. A control condition, in which no SRI intervention occurred, was not included in the present study design. Since the exercise consisted of only moderate intensity exercise, no test–retest interaction is expected, which is consistent with previous investigations utilizing repeated bouts of higher intensity handgrip exercise to evaluate the contributions of specific vasodilators to the forearm blood flow response (Casey et al. 2013).

It is well established that sustained local heating, like that in the present study, produces vasodilation mediated by NO production (Kellogg et al. 1999). This elevation in skin blood flow and vascular conductance is the key response leading to the desired changes in brachial artery blood velocity and shear rate pattern. To ensure the effects of the intervention are due to increased anterograde shear and not the direct effects of heating the muscle or residual NO from the heating, a 15-min recovery period was used to allow the forearm to cool and limb blood flow to return to baseline (Table1). While temperature has the potential to effect endothelial nitric oxide synthase, Venturini et al. (1999) demonstrated that isolated eNOS activity is unaltered by changes in temperature (1999). Likewise, since the half-life of NO within the circulation is <0.1 sec this 15-min period was deemed sufficient to minimize any effects of remaining NO.

**Table 1:** Exercise responses.

	Baseline	Post-SRI	*P*-value
Rest			
MAP (mmHg)	96.3 ± 9.3	92.0 ± 7.9	0.10
Brachial artery diameter (mm)	4.06 ± 0.67	4.06 ± 0.71	0.96
FBF (mL min^−1^)	80.4 ± 26.6	106.6 ± 52.1	0.13
FVC (mL min^−1^ (100 mmHg)^−1^)	85.0 ± 31.6	116.7 ± 56.9	0.11
Steady-state			
MAP (mmHg)	100.6 ± 11.9	96.2 ± 9.2	0.12
Brachial artery diameter (mm)	4.1 ± 0.70	4.26 ± 0.62*	0.03
FBF (mL min^−1^)	251.5 ± 94.4	263.7 ± 83.0	0.45
FVC (mL min^−1^ (100 mmHg)^−1^)	255.4 ± 101.8	277.2 ± 90.3	0.26
Parameter Estimates			
ΔFBF (mL min^−1^)	171.1 ± 70.2	157.6 ± 37.0	0.45
MRT_FBF_, (s)	33.2 ± 16.0	23.0 ± 11.8*	0.02
ΔFVC (mL min^−1^ (100 mmHg)^−1^)	170.3 ± 72.3	160.6 ± 39.0	0.49
MRT_FVC_, (s)	31.1 ± 12.8	20.2 ± 10.8*	0.01

Values are mean ± SD. MAP, mean arterial pressure; FBF, forearm blood flow; FVC, forearm vascular conductance; MRT, mean response time.

* Significantly different from Pre, *P* < 0.05.

### Ultrasound Doppler measurements and calculations

The ultrasound system (Logiq S8; GE Medical Systems, Milwaukee, WI) was operated in duplex mode with a phased linear array transducer probe operated at an imaging frequency of 10.0 MHz, which allowed for simultaneous recording of vessel diameter and blood velocity. The measurement site was approximately midway between the antecubital and axillary regions, medial to *m. biceps brachii*. The location of measurement was marked with indelible ink to ensure similar site of measurement between scans. All Doppler velocity measurements were performed and corrected for an angle of insonation less than 60°. Brachial artery diameter was analyzed at a perpendicular angle along the central axis of the vessel using an automated edge-detection software package (Medical Imaging Applications, Coralville, IA) at a sampling frequency of 15 Hz.

Following the forearm handgrip exercise test brachial artery diameter and blood velocity measurements were time align and used to calculate forearm blood flow (FBF, mL min^−1^) using the product of mean blood velocity (V_mean_, cm sec^−1^) and brachial artery cross-sectional area (cm^2^), which was performed at rest, continuously over each 3 sec contraction cycle for the first 3 min of loaded exercise, and then every 12 sec for the remaining 3 min of exercise. The ratio of FBF and mean arterial pressure (MAP) was used to calculate forearm vascular conductance (FVC, mL min^−1^ (100 mmHg)^−1^). Beat-by-beat MAP was continuously measured via finger photoplethysmography in the contralateral arm (Finometer Pro; Finapress Medical Systems, Amsterdam, The Netherlands) and the mean average calculated across each 3 sec contraction cycle. The beat-by-beat MAP was calibrated to brachial artery blood pressures prior to each exercise test. Resting and steady-state values for brachial artery diameter, FBF, FVC, and MAP were calculated as the average mean of the initial 60 sec rest period prior to exercise and final 60 sec of the exercise period, respectively. ΔFBF and ΔFBC were calculated as the absolute difference between resting and steady-state values.

During the SRI measurements of mean, anterograde, and retrograde shear rate were calculated using mean, anterograde and retrograde blood velocities, respectively. The oscillatory shear index (OSI) was used to characterize the magnitude of the shear rate oscillations throughout a cardiac cycle (Padilla et al. 2010). The OSI was calculated as: OSI = ¦retrograde shear¦/(¦anterograde shear¦ + ¦retrograde shear¦) such that OSI values of zero correspond to a unidirectional shear rate while values of 0.5 are indicative of oscillations with a mean shear rate of zero.

### Kinetics analysis

The kinetics for FBF and FVC were determined by nonlinear least-squares regression using a least squares technique (SigmaPlot 12.5, Systat Software, Point Richmond, CA). With only one transition the overall kinetics for FBF and FVC were described using the mean response time (MRT), which was calculated by fitting a single exponential curve to each response with no time delay from the onset to the end of the handgrip exercise test.

### Statistics

Similar to previous studies investigating the effects of shear rate on vascular responses an 80% power and an *α* of 0.05, at least eight subjects are required to detect a physiologically relevant 2% difference in the steady-state FBF and FVC between conditions. Changes in resting mean, anterograde, and retrograde shear rate pre-, during-, and post-SRI were evaluated with a one-way repeated-measures ANVOA. A Student's paired *t-*test was used to compare the forearm handgrip exercise measurements obtained at baseline and post-SRI. Statistical significance was declared when *P* < 0.05. All tests were conducted using a commercial statistical software package (SigmaPlot/SigmaStat 12.5, Systat Software, Point Richmond, CA). All data are presented as mean ± SD, unless otherwise stated. Given the sample size and need to detect the smallest meaningful physiological differences, effect size comparisons were also made via Cohen's *d* with threshold values for small, moderate, and large effects as 0.2, 0.5, >0.8, respectively(Vincent and Weir 2012).

## Results

**Figure 1:**
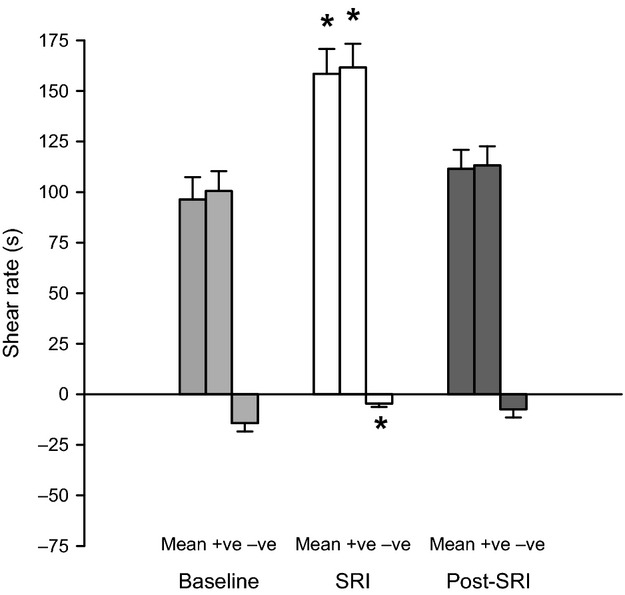
Mean, anterograde (+sr), retrograde (−sr) shear rates in the brachial artery prior to (pre-SRI), and during the shear rate intervention (SRI) elicited via unilateral limb heating. Mean and anterograde shear rate were significantly increased and retrograde shear rate significantly decreased compared to pre-SRI values. The*indicates significant difference versus pre-SRI,* P* < 0.05. Error bars represent SE.

Figure1 illustrates the shear rate patterns elicited at baseline, during the 40 min SRI, and prior to the second forearm handgrip exercise test. During the SRI, mean and anterograde shear rate significantly increased and retrograde shear rate significantly decreased compared to baseline values. These changes resulted in a significant decrease in the OSI of ~79% compared to baseline (0.11 ± 0.07 vs. 0.03 ± 0.03, *P* < 0.05). All shear rate measurements returned back to baseline prior to the start of the second forearm handgrip exercise test.

### Exercise response

**Figure 2:**
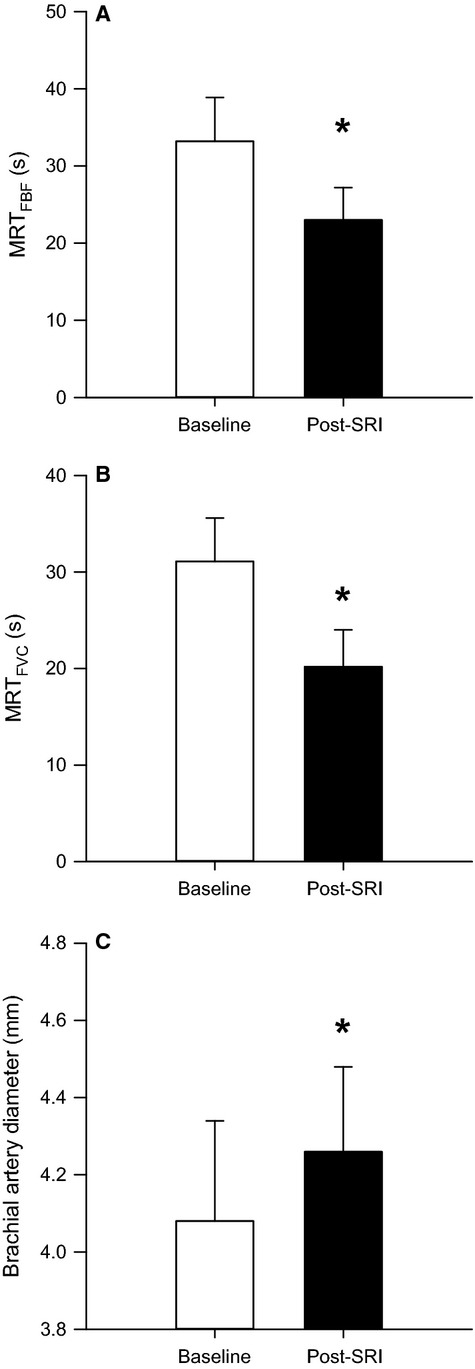
Effects of the 40 min shear rate intervention (SRI) on exercise responses. (A)The post-SRI mean response time for forearm blood flow (MRT_FBF_) and (B) forearm vascular conductance (MRT_FVC_) were significantly faster compared to pre-SRI. (C) The exercise-induced brachial artery vasodilation was significantly greater post- SRI compared to pre-SRI. The *indicates significant difference versus pre-SRI,* P* < 0.05. Error bars represent SE.

The subjects’ MVC were 56.1 ± 8.9 kg (range 46.3–59.2 kg). The mean weight used to achieve 10%MVC for forearm handgrip exercise was 5.61 ± 0.9 kg. There was no significant difference in resting MAP, brachial artery diameter, FBF, or FVC from baseline to post-SRI (Table1). Post-SRI the FBF MRT was significantly faster compared to baseline (Fig.2A). The faster blood flow kinetics at exercise onset post-SRI was achieved in part by a decrease in FVC MRT compared to baseline (Fig.2B). Using Cohen's d, a large effect size was generated for both FBF MRT (*d *=* *1.0) and FVC MRT (*d *=* *1.2). Consistent with faster FVC kinetics, the exercise-induced brachial artery vasodilation was significantly greater after the SRI (Fig. 2C) with a large effect size of 1.0. However, steady-state FBF and FVC were not significantly different between trials with low effect sizes (*d *=* *0.3 and 0.4, respectively).

## Discussion

This investigation is the first to demonstrate that prior exposure to elevated anterograde shear rate alters the vascular responses to dynamic exercise. The primary findings of the present study were that (1) similar to the previous work of Tinken et al. (2009) the SRI, elicited via forearm heating, increased brachial artery mean, and anterograde shear rate, but decreased retrograde shear rate and the OSI; (2) the overall rate of adjustment for FBF and FVC during exercise (post-SRI, measured as MRT) was significantly faster following the 40 min SRI; (3) Post-SRI, the exercise-induced brachial artery diameter was significantly increased compared to baseline. These results support the hypothesis that prior acute exposure to anterograde shear increases the brachial artery vasodilator response to moderate intensity exercise; which supports previous evidence that shear rate can significantly alter vascular function.

Shear stress is the mechanical interaction between blood flow and the endothelial cells lining the arterial wall. It contributes in part to the release of vasoactive substances, the regulation of endothelial gene expression, and over time the structural remodeling of the artery wall (Barakat and Lieu 2003; Newcomer et al. 2011). Experimental evidence from cell culture studies indicate that disturbed or oscillatory flow patterns promote atherosclerosis, while sustained increases in unidirectional laminar flow and shear stress create a pro-vasodilatory and anti-atherosclerotic state (Laughlin et al. 2008; Newcomer et al. 2011). Kuchan and Frangos (1994) demonstrated that exposure of cultured endothelial cells to accelerated laminar fluid flow increased the generation of NO within 30 sec and continued to progress over the next several hours in a shear-dependent manner (Kuchan and Frangos 1994). These data suggest that sustained increases in shear stress ranging from minutes to hours results in a parallel increase in NO availability. Similar studies report that as little as 2–4 h of exposure to an amplified shear stress increase NOS levels, NOS mRNA expression, SOD mRNA expression, and increase the capacity for NO production and release (Ranjan et al. 1995; Uematsu et al. 1995; Woodman et al. 1999). In addition, chronic high blood flow and shear stress produced by arteriovenous fistulas in rat and canine models results in increased NOS mRNA expression, NOS protein levels, and endothelium-dependent relaxation (Miller and Burnett 1992; Nadaud et al. 1996; Jeon et al. 2000).

Unlike cell culture models, the intact cardiovascular system at rest exhibits a phasic flow pattern in peripheral conduit arteries such that during systole a large anterograde flow is followed by a period of retrograde flow of varying degrees that is dependent on resting vascular tone (McDonald 1955; Ade et al. 2012). Similar to the present investigation Tinken et al. (2009) acutely modified in vivo shear stress and demonstrated that following a 30-min shear rate intervention, endothelial function, as measured by brachial artery endothelium-dependent flow-mediated dilation, was significantly increased (Tinken et al. 2009). The present investigation expands on this earlier investigation to include increases in FBF and FVC kinetics and the exercise-induced arterial dilation.

The magnitude and time course of the increase in blood flow during dynamic exercise are dependent on the integrated actions of metabolic control, the muscle-pump, myogenic vasodilation, and flow-induced endothelium-mediated vasodilation (Delp and Laughlin 1998; Saltin et al. 1998; Clifford and Hellsten 2004; Wray et al. 2011). Specific to dynamic handgrip exercise like that used in the present investigation, Wray et al. (2011) demonstrated a NO-mediated component to the brachial artery dilation and FBF response to exercise, particularly at higher workloads(Wray et al. 2011). This is consistent with early work that showed that the conduit artery response to exercise is dependent upon work rate and that several minutes may be required for FBF to reach steady state(Shoemaker et al. 1997b). Recent work by Casey et al. (2013) further evaluated the contribution of NO to the on-transient increase in vascular conductance during forearm exercise in humans (2013). Following NOS inhibition the rate of vasodilation was decreased and the time to reach a steady state was increased compared to control conditions. However, this conflicts with the findings of Shoemaker et al. (1997a,b) who suggest neither acetylcholine nor NO contribute to the rate of increases for FBF during handgrip exercise(Shoemaker et al. 1997a). In the present study, prior exposure to an increase in shear rate resulted in significantly faster on-transient FVC and FBF responses during exercise in the brachial artery. Likewise, exercise-induced brachial artery diameter was significantly increased post-SRI compared to pre-SRI. These results in total provide some evidence that prior exposure to increases in anterograde shear rate results in positive vascular alterations during exercise, potentially through some endothelial-mediated pathway.

### Experimental considerations

Several methodological considerations are relevant to the interpretation of the present investigation. First, measurements of shear rate in the brachial artery may be different than what occurred downstream in the resistance arterioles. Since measurements of shear rate within these vessels could not be performed in the present study, brachial artery shear rate was used as a broad measurement of the shear stimulus and therefore should be interpreted appropriately. This may explain in part the significant increase in exercise-induced brachial artery dilation, but not steady-state FBF or FVC. Second, the present study did not attempt to evaluate the contribution of individual vasoactive substances (i.e., NO, adenosine, prostaglandins) via pharmaceutical blockade. While this limits identification of specific mechanisms contributing to the enhanced vasodilator responses observed following the shear rate intervention, it does not diminish the primary findings of the present study. However, previous work has demonstrated a strong NO-dependent mechanism for the on-transient and steady-state increase in vascular conductance during exercise (Ferreira et al. 2006; Wray et al. 2011; Casey et al. 2013). Forearm heating was also used to generate an increase in brachial artery anterograde shear rate. The residual effects of an elevated, skin temperature, and presumably skin blood flow, was controlled for by inclusion of a 15 min postheating period to allow for return to preheating values, which is supported by no significant difference between baseline brachial artery diameter, FBF, or FVC (Table1). In addition, any residual effects on endothelial nitric oxide synthase and NO should have subsided, given that eNOS activity is unaltered by changes in temperature and half-life of NO within the circulation is <0.1 sec (Venturini et al. 1999). Lastly, the study did not include a control condition or arm. It is possible that the reported changes are due to the first bout of exercise. Since the exercise consisted of only moderate intensity exercise little to no test–retest interaction is expected as is consistent with previous investigations utilizing repeated bouts of handgrip exercise (Casey et al. 2013). Therefore, it is unlikely, that the change is due to the changes in exercise responses over time.

### Implications and conclusions

This study investigated the effects of an acute increase in shear rate on vascular function during dynamic handgrip exercise. FBF and FVC kinetics at exercise onset are dependent on the rate at which the endothelium and vascular smooth muscle can response to a given exercise stimulus. In young healthy men, faster blood flow kinetics may only be associated with improved vascular function, which may or may not result in health or exercise performance implications. However, if blood flow kinetics can be improved in certain populations, like the elderly or diseased, the faster O_2_ delivery at exercise onset would translate to faster VO_2_ onset kinetics, which would minimize intracellular perturbations (e.g., Δ[H+], Δ[lacate], Δ[PCr]) and improve exercise tolerance (Poole and Jones 2012). The present study demonstrated that prior exposure to a high anterograde shear rate accelerates the on-transient increase in FBF and FVC during moderate intensity exercise and increased exercise-induced brachial artery vasodilation in healthy young men. These findings highlight a key point, that anterograde shear rate patterns significantly improve the rate at which the vascular responds to a mild exercise stress, which in future studies may provide a means of improving vascular function and O_2_ uptake kinetics in certain populations. However, further investigation is required to determine if similar results are observed in the elderly or cardiovascular disease populations. In conclusion, these results highlight and extend our current understanding of the possible beneficial effects of prolonged exposure to anterograde shear rate in vivo.
